# Bis­(thio­cyanato-κ*N*)[tris(pyridin-2-yl­meth­yl)amine-κ^4^ *N*]­iron(II)

**DOI:** 10.1107/S1600536813034818

**Published:** 2014-01-08

**Authors:** Jing-Wei Dai, Zhao-Yang Li, Osamu Sato

**Affiliations:** a Institute for Materials Chemistry and Engineering, Kyushu University, 6-1 Kasuga-koen, Kasuga, Fukuoka 816-8580, Japan; b Department of Chemistry, Graduate School of Science, Tohoku University, 6-3 Aramaki-Aza-Aoba, Aoba-ku, Sendai 980-8578, Japan

## Abstract

In the title complex, [Fe(NCS)_2_(C_18_H_18_N_4_)], the Fe^II^ cation is chelated by a tris­(2-pyridyl­meth­yl)amine ligand and coordin­ated by two thio­cyanate anions in a distorted N_6_ octa­hedral geometry. In the crystal, weak C—H⋯S hydrogen bonds and π–π stacking inter­actions between parallel pyridine rings of adjacent mol­ecules [centroid–centroid distance = 3.653 (3) Å] link the mol­ecules into a two-dimensional supra­molecular architecture. The structure contains voids of 124 (9) Å^3^, which are free of solvent molecules.

## Related literature   

For the magnetic properties of metal complexes with tris­(2-pyridyl­meth­yl)amine and thio­cyanate ligands, see: Boldog *et al.* (2009 ▶); Li *et al.* (2010 ▶). For related complexes, see: Benhamou *et al.* (2008 ▶); Min *et al.* (2008 ▶); Phan *et al.* (2012 ▶); Wei *et al.* (2011 ▶).
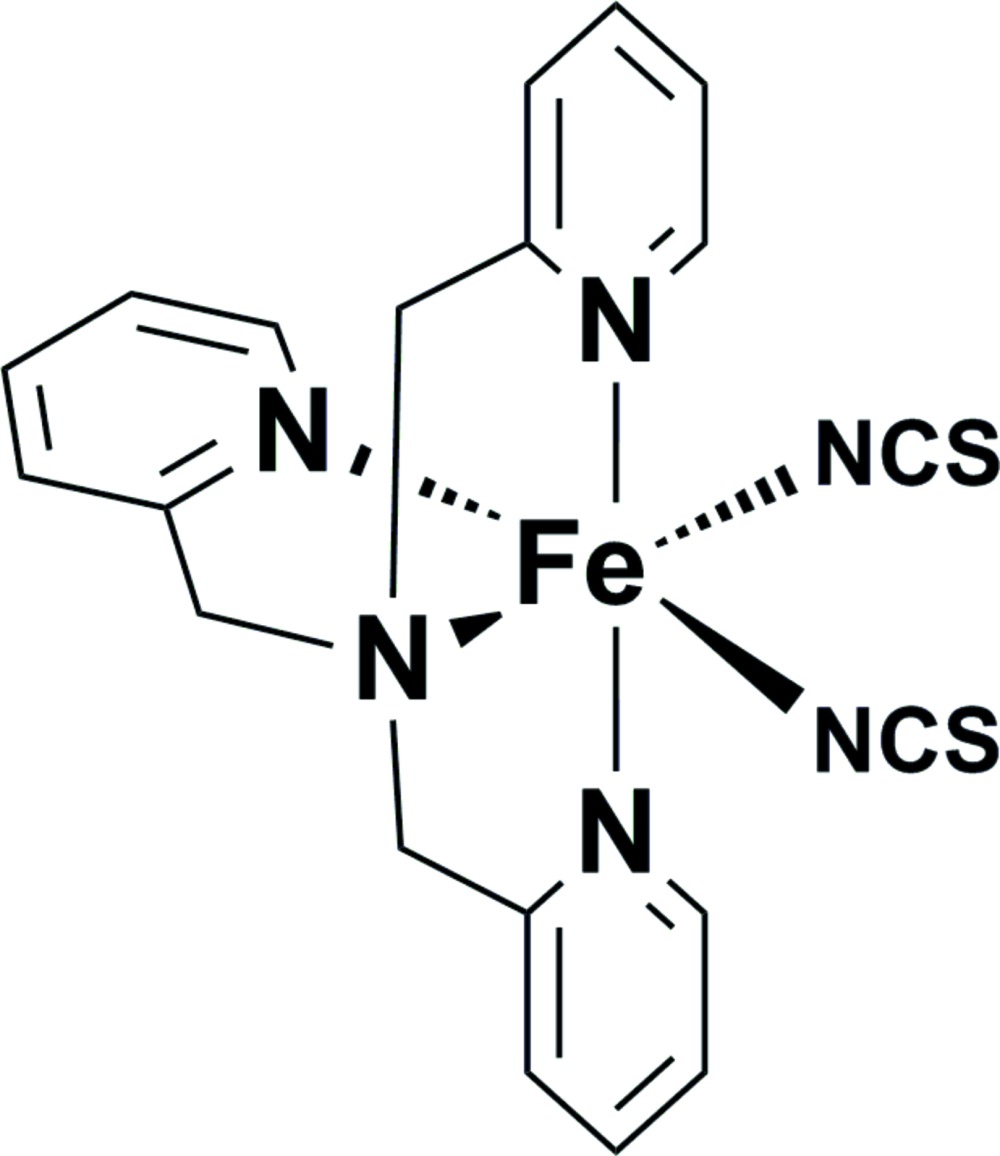


## Experimental   

### 

#### Crystal data  

[Fe(NCS)_2_(C_18_H_18_N_4_)]
*M* *_r_* = 462.37
Monoclinic, 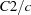

*a* = 23.714 (5) Å
*b* = 11.827 (2) Å
*c* = 17.580 (3) Å
β = 112.87 (3)°
*V* = 4543.0 (18) Å^3^
*Z* = 8
Mo *K*α radiation
μ = 0.87 mm^−1^
*T* = 123 K
0.20 × 0.20 × 0.10 mm

#### Data collection  

Rigaku Saturn70 diffractometer
Absorption correction: multi-scan (*CrystalClear*; Rigaku, 2008 ▶) *T*_min_ = 0.84, *T*_max_ = 0.92
10256 measured reflections
4456 independent reflections
3275 reflections with *I* > 2σ(*I*)
*R*_int_ = 0.059

#### Refinement  

*R*[*F* ^2^ > 2σ(*F* ^2^)] = 0.069
*wR*(*F* ^2^) = 0.203
*S* = 1.11
4456 reflections
283 parameters
H atoms treated by a mixture of independent and constrained refinement
Δρ_max_ = 0.42 e Å^−3^
Δρ_min_ = −0.47 e Å^−3^

### 

Data collection: *CrystalClear* (Rigaku, 2008 ▶); cell refinement: *CrystalClear*; data reduction: *CrystalClear*; program(s) used to solve structure: *SHELXTL* (Sheldrick, 2008 ▶); program(s) used to refine structure: *SHELXTL*; molecular graphics: *SHELXTL*; software used to prepare material for publication: *SHELXTL*.

## Supplementary Material

Crystal structure: contains datablock(s) I, global. DOI: 10.1107/S1600536813034818/xu5762sup1.cif

Structure factors: contains datablock(s) I. DOI: 10.1107/S1600536813034818/xu5762Isup2.hkl

CCDC reference:

Additional supporting information:  crystallographic information; 3D view; checkCIF report

## Figures and Tables

**Table 1:** Selected bond lengths (Å)

Fe1—N1	2.185 (4)
Fe1—N2	2.197 (4)
Fe1—N3	2.199 (4)
Fe1—N4	2.241 (4)
Fe1—N5	2.054 (5)
Fe1—N6	2.089 (4)

**Table 2:** Hydrogen-bond geometry (Å, °)

*D*—H⋯*A*	*D*—H	H⋯*A*	*D*⋯*A*	*D*—H⋯*A*
C4—H4⋯S2^i^	0.95	2.98	3.725 (6)	136
C12—H12*B*⋯S2^ii^	0.99	2.89	3.850 (5)	164
C18—H18*B*⋯S1^ii^	0.99	2.97	3.635 (5)	126

Symmetry codes: (i) 
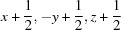
; (ii) 
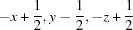
.

**Fig. 1.**
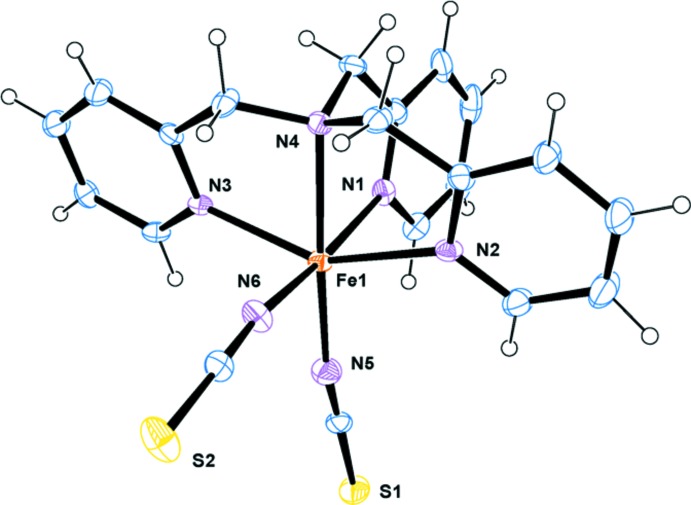
Crystal structure of the title compound with labelling and displacement ellipsoids drawn at the 30% probability level.

**Fig. 2.**
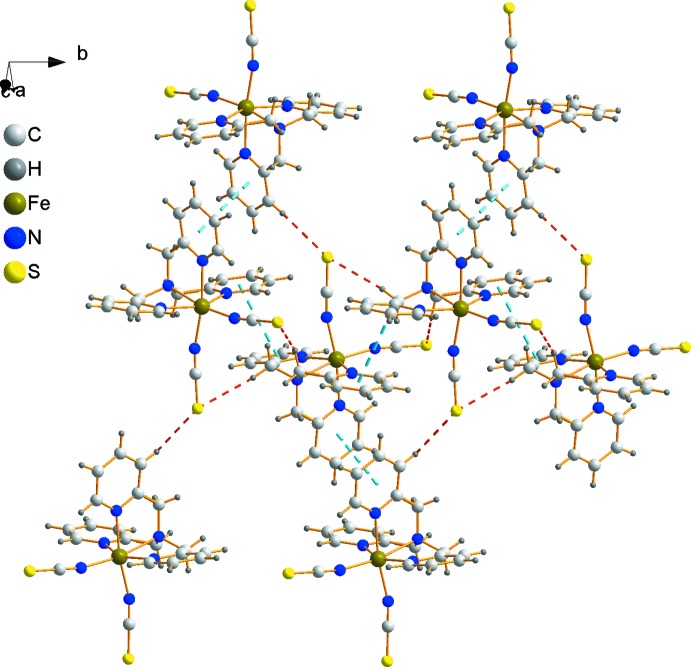
Crystal packing of the title complex. Intermolecular C—H···S hydrogen bonds are shown as red dashed lines, and π–π stacking interactions between pyridine rings are shown as blue dashed lines.

## supplementary crystallographic information

### 1. Comment

A series of iron complexes with the tris(2-pyridylmethyl)amine (tpa) ligands (Benhamou *et al.*, 2008; Boldog *et al.*, 2009; Li *et al.*, 2010; Min *et al.*, 2008; Phan *et al.*, 2012; Wei *et al.*, 2011) that exhibit interesting magnetic properties have been synthesized and structurally characterized. In these complexes, the tpa ligands coordinate to the metal centers through its pyridyl nitrogen atoms and arylamine nitrogen atom. It should be noted that the magnetic behavior of the Fe(II) spin crossover complex with the ligand tpa and thiocyanato has already been reported (Boldog *et al.*, 2009; Li *et al.*, 2010).

In the title compound, Fe(C_18_H_18_N_4_)(NCS)_2_, the Fe(II) atom is coordinated by six nitrogen atoms (Fig. 1). The Fe-N distances range from 2.054 (5) to 2.241 (4) Å. In the crystal, π-π stacking 3.653 (3) Å between parallel pyridine rings and weak C—H···S hydrogen bonds link the molecules into the two dimensional supramolecular structure (Fig. 2).

### 2. Experimental

A mixture of FeSO_4_.7H_2_O (0.0281 g, 0.1 mmol), tris(2-pyridylmethyl)amine (tpa, 0.0291 g, 0.1 mmol), KSCN (0.0194 g, 0.2 mmol) and MeOH (5 mL) were sealed in a 25 mL Teflon reactor and heated at 160^o^C for 48 h, and then cooled to ambient temperature at a rate of *ca.* 10 ^o^C h^-1^ to give yellow block crystals (yield: 21%, based on tpa).

### 3. Refinement

H2, H7, H8, H14, H16, H16A and H16B atoms were located in a difference Fourier map and refined isotropicaly with U_iso_(H) = 0.026 Å^2^. Other H atoms were placed at calculated positions and were treated as riding on the parent C atoms with C—H = 0.95–0.99 Å, U_iso_(H) = 1.2U_eq_(C).

### Crystal data

**Table:**

[Fe(NCS)_2_(C_18_H_18_N_4_)]	*F*(000) = 1904
*M**_r_* = 462.37	*D*_x_ = 1.352 Mg m^−^^3^
Monoclinic, *C*2/*c*	Mo *K*α radiation, λ = 0.71073 Å
Hall symbol: -C 2yc	Cell parameters from 5300 reflections
*a* = 23.714 (5) Å	θ = 3.1–27.5°
*b* = 11.827 (2) Å	µ = 0.87 mm^−^^1^
*c* = 17.580 (3) Å	*T* = 123 K
β = 112.87 (3)°	Block, yellow
*V* = 4543.0 (18) Å^3^	0.20 × 0.20 × 0.10 mm
*Z* = 8	

### Data collection

**Table:**

Rigaku Saturn70 diffractometer	4456 independent reflections
Radiation source: Rotating Anode	3275 reflections with *I* > 2σ(*I*)
Confocal monochromator	*R*_int_ = 0.059
Detector resolution: 28.5714 pixels mm^-1^	θ_max_ = 26.0°, θ_min_ = 3.1°
dtprofit.ref scans	*h* = −29→28
Absorption correction: multi-scan (*CrystalClear*; Rigaku, 2008)	*k* = −13→14
*T*_min_ = 0.84, *T*_max_ = 0.92	*l* = −21→15
10256 measured reflections	

### Refinement

**Table:**

Refinement on *F*^2^	Primary atom site location: structure-invariant direct methods
Least-squares matrix: full	Secondary atom site location: difference Fourier map
*R*[*F*^2^ > 2σ(*F*^2^)] = 0.069	Hydrogen site location: inferred from neighbouring sites
*wR*(*F*^2^) = 0.203	H atoms treated by a mixture of independent and constrained refinement
*S* = 1.11	*w* = 1/[σ^2^(*F*_o_^2^) + (0.0906*P*)^2^ + 9.7621*P*] where *P* = (*F*_o_^2^ + 2*F*_c_^2^)/3
4456 reflections	(Δ/σ)_max_ < 0.001
283 parameters	Δρ_max_ = 0.42 e Å^−^^3^
0 restraints	Δρ_min_ = −0.47 e Å^−^^3^

### Special details

**Table:**

Geometry. All e.s.d.'s (except the e.s.d. in the dihedral angle between two l.s. planes) are estimated using the full covariance matrix. The cell e.s.d.'s are taken into account individually in the estimation of e.s.d.'s in distances, angles and torsion angles; correlations between e.s.d.'s in cell parameters are only used when they are defined by crystal symmetry. An approximate (isotropic) treatment of cell e.s.d.'s is used for estimating e.s.d.'s involving l.s. planes.
Refinement. Refinement of *F*^2^ against ALL reflections. The weighted *R*-factor *wR* and goodness of fit *S* are based on *F*^2^, conventional *R*-factors *R* are based on *F*, with *F* set to zero for negative *F*^2^. The threshold expression of *F*^2^ > σ(*F*^2^) is used only for calculating *R*-factors(gt) *etc*. and is not relevant to the choice of reflections for refinement. *R*-factors based on *F*^2^ are statistically about twice as large as those based on *F*, and *R*- factors based on ALL data will be even larger.

### Fractional atomic coordinates and isotropic or equivalent isotropic displacement parameters (Å^2^)

**Table:**

	*x*	*y*	*z*	*U*_iso_*/*U*_eq_	
C1	0.4648 (2)	0.4925 (4)	0.3477 (3)	0.0314 (12)	
H1	0.4527	0.5481	0.3053	0.038*	
C2	0.5264 (3)	0.4840 (6)	0.3995 (4)	0.0453 (16)	
C3	0.5440 (3)	0.4028 (6)	0.4600 (4)	0.0500 (18)	
H3	0.5858	0.3957	0.4960	0.060*	
C4	0.5011 (3)	0.3321 (5)	0.4683 (4)	0.0442 (15)	
H4	0.5126	0.2749	0.5095	0.053*	
C5	0.4394 (2)	0.3459 (4)	0.4144 (3)	0.0291 (11)	
C6	0.3910 (3)	0.2752 (5)	0.4272 (3)	0.0308 (12)	
C7	0.3845 (3)	0.2760 (5)	0.1662 (4)	0.0365 (13)	
C8	0.4054 (3)	0.1794 (5)	0.1395 (4)	0.0515 (18)	
C9	0.3991 (3)	0.0772 (5)	0.1708 (4)	0.0537 (19)	
H9	0.4129	0.0104	0.1534	0.064*	
C10	0.3727 (3)	0.0710 (4)	0.2277 (4)	0.0394 (14)	
H10	0.3684	0.0003	0.2507	0.047*	
C11	0.3524 (2)	0.1698 (4)	0.2509 (3)	0.0269 (11)	
C12	0.3195 (2)	0.1693 (4)	0.3090 (3)	0.0301 (12)	
H12A	0.2750	0.1604	0.2770	0.036*	
H12B	0.3337	0.1043	0.3473	0.036*	
C13	0.3010 (2)	0.6046 (4)	0.3840 (3)	0.0268 (11)	
H13	0.3135	0.6574	0.3528	0.032*	
C14	0.2846 (3)	0.6447 (4)	0.4460 (4)	0.0334 (13)	
C15	0.2664 (3)	0.5696 (4)	0.4917 (4)	0.0368 (13)	
H15	0.2550	0.5950	0.5349	0.044*	
C16	0.2654 (3)	0.4564 (5)	0.4731 (4)	0.0374 (14)	
C17	0.2822 (2)	0.4211 (4)	0.4102 (3)	0.0239 (10)	
C18	0.2812 (3)	0.2989 (4)	0.3860 (4)	0.0322 (12)	
H18A	0.2862	0.2501	0.4340	0.039*	
H18B	0.2412	0.2809	0.3414	0.039*	
C19	0.3519 (2)	0.6301 (4)	0.1751 (3)	0.0280 (11)	
C20	0.1843 (2)	0.3861 (4)	0.1455 (3)	0.0283 (11)	
Fe1	0.32597 (3)	0.41694 (5)	0.26973 (4)	0.0221 (2)	
H2	0.550 (2)	0.542 (4)	0.396 (3)	0.026*	
H7	0.391 (2)	0.349 (5)	0.147 (3)	0.026*	
H8	0.416 (2)	0.182 (4)	0.092 (3)	0.026*	
H14	0.287 (2)	0.726 (4)	0.459 (3)	0.026*	
H16	0.248 (2)	0.408 (4)	0.495 (3)	0.026*	
H6A	0.405 (2)	0.203 (5)	0.442 (3)	0.026*	
H6B	0.382 (2)	0.298 (4)	0.469 (3)	0.026*	
N1	0.42170 (18)	0.4244 (3)	0.3555 (3)	0.0259 (9)	
N2	0.35696 (18)	0.2708 (3)	0.2191 (3)	0.0256 (9)	
N3	0.30029 (17)	0.4938 (3)	0.3656 (2)	0.0201 (8)	
N4	0.33090 (18)	0.2760 (3)	0.3573 (3)	0.0240 (9)	
N5	0.3355 (2)	0.5542 (4)	0.2041 (3)	0.0376 (11)	
N6	0.23443 (19)	0.3841 (3)	0.1954 (3)	0.0303 (10)	
S1	0.37451 (7)	0.73656 (12)	0.13592 (10)	0.0415 (4)	
S2	0.11437 (7)	0.39138 (13)	0.07738 (11)	0.0517 (5)	

### Atomic displacement parameters (Å^2^)

**Table:**

	*U*^11^	*U*^22^	*U*^33^	*U*^12^	*U*^13^	*U*^23^
C1	0.029 (3)	0.035 (3)	0.030 (3)	−0.009 (2)	0.012 (2)	−0.006 (2)
C2	0.026 (3)	0.060 (4)	0.048 (4)	−0.013 (3)	0.013 (3)	−0.030 (3)
C3	0.023 (3)	0.081 (5)	0.042 (4)	0.005 (3)	0.008 (3)	−0.028 (4)
C4	0.032 (3)	0.060 (4)	0.034 (3)	0.021 (3)	0.007 (3)	−0.008 (3)
C5	0.029 (3)	0.032 (3)	0.023 (3)	0.010 (2)	0.007 (2)	−0.004 (2)
C6	0.040 (3)	0.028 (3)	0.022 (3)	0.010 (2)	0.010 (2)	0.007 (2)
C7	0.049 (3)	0.031 (3)	0.033 (3)	0.000 (2)	0.021 (3)	0.002 (2)
C8	0.084 (5)	0.038 (3)	0.054 (4)	0.001 (3)	0.050 (4)	−0.003 (3)
C9	0.090 (5)	0.030 (3)	0.063 (5)	0.015 (3)	0.053 (4)	0.003 (3)
C10	0.060 (4)	0.025 (3)	0.044 (4)	0.002 (3)	0.033 (3)	−0.003 (2)
C11	0.034 (3)	0.021 (2)	0.026 (3)	0.001 (2)	0.011 (2)	−0.002 (2)
C12	0.043 (3)	0.020 (2)	0.032 (3)	−0.002 (2)	0.019 (3)	0.002 (2)
C13	0.033 (3)	0.021 (2)	0.025 (3)	−0.002 (2)	0.009 (2)	0.006 (2)
C14	0.042 (3)	0.019 (3)	0.042 (3)	0.009 (2)	0.019 (3)	−0.001 (2)
C15	0.053 (4)	0.027 (3)	0.038 (3)	0.008 (2)	0.026 (3)	0.002 (2)
C16	0.053 (4)	0.025 (3)	0.046 (4)	0.005 (2)	0.032 (3)	0.008 (3)
C17	0.026 (3)	0.019 (2)	0.025 (3)	0.0048 (19)	0.008 (2)	0.003 (2)
C18	0.048 (3)	0.020 (2)	0.042 (3)	−0.006 (2)	0.032 (3)	0.001 (2)
C19	0.032 (3)	0.031 (3)	0.018 (3)	0.003 (2)	0.005 (2)	−0.001 (2)
C20	0.034 (3)	0.018 (2)	0.032 (3)	−0.001 (2)	0.012 (3)	−0.006 (2)
Fe1	0.0252 (4)	0.0184 (4)	0.0213 (4)	−0.0012 (3)	0.0075 (3)	0.0002 (3)
N1	0.024 (2)	0.027 (2)	0.024 (2)	−0.0009 (17)	0.0062 (18)	−0.0084 (18)
N2	0.034 (2)	0.0190 (19)	0.023 (2)	−0.0005 (17)	0.0102 (19)	0.0027 (17)
N3	0.0198 (19)	0.0190 (19)	0.018 (2)	−0.0029 (15)	0.0035 (16)	0.0009 (16)
N4	0.029 (2)	0.0165 (19)	0.028 (2)	0.0000 (16)	0.0131 (19)	0.0017 (17)
N5	0.044 (3)	0.033 (2)	0.038 (3)	0.002 (2)	0.017 (2)	0.004 (2)
N6	0.028 (2)	0.022 (2)	0.032 (3)	−0.0008 (17)	0.002 (2)	−0.0096 (18)
S1	0.0507 (9)	0.0343 (8)	0.0475 (9)	−0.0017 (6)	0.0279 (8)	0.0099 (7)
S2	0.0352 (9)	0.0469 (9)	0.0526 (11)	0.0060 (7)	−0.0051 (8)	−0.0199 (8)

### Geometric parameters (Å, º)

**Table:**

C1—N1	1.350 (6)	C12—H12A	0.9900
C1—C2	1.393 (8)	C12—H12B	0.9900
C1—H1	0.9500	C13—N3	1.349 (6)
C2—C3	1.372 (10)	C13—C14	1.377 (7)
C2—H2	0.91 (5)	C13—H13	0.9500
C3—C4	1.367 (9)	C14—C15	1.373 (8)
C3—H3	0.9500	C14—H14	0.98 (5)
C4—C5	1.409 (7)	C15—C16	1.376 (7)
C4—H4	0.9500	C15—H15	0.9500
C5—N1	1.332 (7)	C16—C17	1.378 (7)
C5—C6	1.506 (7)	C16—H16	0.87 (5)
C6—N4	1.476 (6)	C17—N3	1.341 (6)
C6—H6A	0.91 (5)	C17—C18	1.504 (6)
C6—H6B	0.88 (5)	C18—N4	1.477 (6)
C7—N2	1.330 (7)	C18—H18A	0.9900
C7—C8	1.396 (8)	C18—H18B	0.9900
C7—H7	0.96 (5)	C19—N5	1.172 (7)
C8—C9	1.361 (9)	C19—S1	1.623 (5)
C8—H8	0.97 (5)	C20—N6	1.172 (7)
C9—C10	1.373 (8)	C20—S2	1.626 (6)
C9—H9	0.9500	Fe1—N1	2.185 (4)
C10—C11	1.385 (7)	Fe1—N2	2.197 (4)
C10—H10	0.9500	Fe1—N3	2.199 (4)
C11—N2	1.341 (6)	Fe1—N4	2.241 (4)
C11—C12	1.509 (7)	Fe1—N5	2.054 (5)
C12—N4	1.485 (6)	Fe1—N6	2.089 (4)
			
N1—C1—C2	122.2 (5)	C14—C15—H15	120.9
N1—C1—H1	118.9	C16—C15—H15	120.9
C2—C1—H1	118.9	C15—C16—C17	120.0 (5)
C3—C2—C1	118.8 (6)	C15—C16—H16	120 (3)
C3—C2—H2	125 (4)	C17—C16—H16	119 (3)
C1—C2—H2	115 (4)	N3—C17—C16	122.3 (4)
C4—C3—C2	119.8 (6)	N3—C17—C18	115.1 (4)
C4—C3—H3	120.1	C16—C17—C18	122.6 (4)
C2—C3—H3	120.1	N4—C18—C17	110.2 (4)
C3—C4—C5	118.6 (6)	N4—C18—H18A	109.6
C3—C4—H4	120.7	C17—C18—H18A	109.6
C5—C4—H4	120.7	N4—C18—H18B	109.6
N1—C5—C4	122.3 (5)	C17—C18—H18B	109.6
N1—C5—C6	118.3 (4)	H18A—C18—H18B	108.1
C4—C5—C6	119.2 (5)	N5—C19—S1	179.1 (5)
N4—C6—C5	114.7 (4)	N6—C20—S2	178.7 (5)
N4—C6—H6A	111 (3)	N5—Fe1—N6	96.34 (18)
C5—C6—H6A	111 (3)	N5—Fe1—N1	92.51 (18)
N4—C6—H6B	103 (3)	N6—Fe1—N1	170.87 (16)
C5—C6—H6B	113 (3)	N5—Fe1—N2	105.47 (17)
H6A—C6—H6B	102 (5)	N6—Fe1—N2	91.73 (15)
N2—C7—C8	122.2 (5)	N1—Fe1—N2	83.71 (15)
N2—C7—H7	119 (3)	N5—Fe1—N3	103.16 (16)
C8—C7—H7	119 (3)	N6—Fe1—N3	91.49 (15)
C9—C8—C7	118.8 (6)	N1—Fe1—N3	88.68 (14)
C9—C8—H8	119 (3)	N2—Fe1—N3	150.64 (14)
C7—C8—H8	121 (3)	N5—Fe1—N4	170.32 (17)
C8—C9—C10	119.7 (5)	N6—Fe1—N4	93.18 (16)
C8—C9—H9	120.2	N1—Fe1—N4	78.06 (15)
C10—C9—H9	120.2	N2—Fe1—N4	75.94 (14)
C9—C10—C11	118.6 (5)	N3—Fe1—N4	74.74 (14)
C9—C10—H10	120.7	C5—N1—C1	118.2 (5)
C11—C10—H10	120.7	C5—N1—Fe1	116.0 (3)
N2—C11—C10	122.4 (5)	C1—N1—Fe1	125.3 (4)
N2—C11—C12	115.6 (4)	C7—N2—C11	118.3 (4)
C10—C11—C12	121.8 (4)	C7—N2—Fe1	125.5 (3)
N4—C12—C11	110.9 (4)	C11—N2—Fe1	116.0 (3)
N4—C12—H12A	109.5	C17—N3—C13	117.3 (4)
C11—C12—H12A	109.5	C17—N3—Fe1	115.5 (3)
N4—C12—H12B	109.5	C13—N3—Fe1	127.2 (3)
C11—C12—H12B	109.5	C6—N4—C18	111.0 (4)
H12A—C12—H12B	108.1	C6—N4—C12	111.9 (4)
N3—C13—C14	122.9 (4)	C18—N4—C12	111.0 (4)
N3—C13—H13	118.5	C6—N4—Fe1	110.6 (3)
C14—C13—H13	118.5	C18—N4—Fe1	105.3 (3)
C15—C14—C13	119.3 (5)	C12—N4—Fe1	106.9 (3)
C15—C14—H14	120 (3)	C19—N5—Fe1	168.1 (4)
C13—C14—H14	121 (3)	C20—N6—Fe1	165.9 (4)
C14—C15—C16	118.2 (5)		

### Hydrogen-bond geometry (Å, º)

**Table:**

*D*—H···*A*	*D*—H	H···*A*	*D*···*A*	*D*—H···*A*
C4—H4···S2^i^	0.95	2.98	3.725 (6)	136
C12—H12*B*···S2^ii^	0.99	2.89	3.850 (5)	164
C18—H18*B*···S1^ii^	0.99	2.97	3.635 (5)	126

Symmetry codes: (i) *x*+1/2, −*y*+1/2, *z*+1/2; (ii) −*x*+1/2, *y*−1/2, −*z*+1/2.
